# Apheresis: What Should a Clinician Know?

**DOI:** 10.1007/s11883-023-01081-7

**Published:** 2023-01-26

**Authors:** Klaus G. Parhofer

**Affiliations:** Medical Department IV – Grosshadern, University Munich, Marchioninistraße 15, 81377 Munich, Germany

**Keywords:** Lipoprotein apheresis, Lipoprotein(a), Dyslipoproteinemia, Familial hypercholesterolemia

## Abstract

**Purpose of Review:**

Apheresis is a treatment option for severe dyslipidemia which has been introduced approximately 40 years ago to clinical practice. This article reviews recent apheresis research progresses, including apheresis for elevated LDL-cholesterol and elevated lipoprotein(a).

**Recent Findings:**

While the role of apheresis in treating more common forms of LDL-hypercholesterolemia has been reduced due to the development of new, very potent LDL-lowering drugs, it still plays an important role in treating patients with homozygous familial hypercholesterolemia and patients with severe lipoprotein(a) elevation. One apheresis session can decrease LDL-cholesterol, apoB, and lipoprotein(a) by approximately 65%, which results in a time averaged reduction of 30–50%. Although time-consuming, and expensive regular apheresis is very well tolerated and has been proven safe for decades.

**Summary:**

Apheresis remains a treatment option for severe dyslipidemia, especially in homozygous familial hypercholesterolemia and elevated lipoprotein(a), if other forms of therapy fail to achieve targets.

## Introduction

Elevated concentrations of lipoproteins are directly and causally linked to atherosclerosis [1–3]. Reducing LDL-cholesterol and the concentrations of other atherogenic lipoproteins is therefore an important strategy to prevent cardiovascular disease. Lipid lowering drugs such as statins, ezetimibe, and proprotein convertase subtilisin/kexin type 9 (PCSK9) inhibitors have been shown to reduce cardiovascular event rates by modifying plasma lipoproteins [3]. Based on a very convincing set of data, guidelines have been published which state lipoprotein goals depending on the overall cardiovascular risk. In most patients, an escalation strategy is used to reach the LDL-cholesterol goals, which includes life style modification, statins, ezetimibe, bempedoic acid, and PCSK9 inhibitors such as alirocumab, evolocumab, and inclisiran. However, some patients cannot achieve these goals either because they are intolerant to the abovementioned medications or more often because the underlying dyslipidemia is resistant to drug therapy (for example, homozygous familial hypercholesterolemia or elevated lipoprotein(a)).

For more than 40 years, apheresis has been a therapy of last resort to address dyslipoproteinemias that can otherwise not be treated [4]. However, the development of new medications, such as PCSK9 inhibitors, has shifted the indications, as more forms of dyslipidemia then before can be treated nowadays without apheresis.

Apheresis was first described as a treatment option for homozygous familial hypercholesterolemia in 1975 [4]. Later, more specific techniques were developed to specifically reduce apoB containing particles such as LDL and lipoprotein(a) [5]. While the focus of apheresis has been the reduction of LDL until 2015 (when PCSK9 antibodies were introduced in the market), the indication has now shifted more toward elevated lipoprotein(a).

Currently five different apheresis techniques are available to eliminate apolipoprotein-B containing lipoproteins. In addition, there is one technique specific for lipoprotein(a) reduction.

In this review, the current role of apheresis in the treatment of dyslipidemia is discussed, considering the currently available drugs.

## Apheresis for Elevated LDL-Cholesterol

In most patients with LDL-hypercholesterolemia, with the exception of some patients with severe heterozygous familial hypercholesterolemia or homozygous familial Hypercholesterolemia and some patients with statin intolerance, LDL-cholesterol goals can be achieved if a combination of lifestyle modification, statins, ezetimibe, bempedoic acid, and PCSK9 inhibitors is used [3]. Particularly, the availability of PCSK9 inhibitors has dramatically reduced the number of patients requiring apheresis for elevated LDL-cholesterol, as patients with familial hypercholesterolemia usually respond well to PCSK9 inhibitor therapy and the majority of patients with statin intolerance tolerate PCSK9 inhibitors well [6]. Therefore, in patients on regular apheresis, the use of PCSK9 inhibitors allows to stop apheresis therapy in 63.4% and allows to lengthen intervals between treatments in 92.7% [7]. Overall PCSK9 inhibitors decrease LDL-cholesterol to a similar extent as apheresis. While inflammatory parameters are decreased with long-term apheresis, these parameters were not influenced by PCSK9 inhibitors [8, 9]. Although the effect of apheresis and PCSK9 inhibition is similar with respect to LDL-cholesterol reduction, apheresis eliminates additional molecules, and it is unknown to what extent these pleiotropic effects of apheresis determine the clinical benefit.

Indications for apheresis for elevated LDL-cholesterol considerably vary by country [10]. In the USA, homozygous familial hypercholesterolemia is the main indication for lipoprotein apheresis. It is also approved for patients with other severe forms LDL-hypercholesterolemia which persist despite maximal drug therapy (LDL-cholesterol > 300 mg/dl without concomitant cardio-vascular disease or > 200 mg/dl with concomitant cardiovascular disease) [11]. In Germany, apheresis for elevated LDL-cholesterol can be performed in severe hypercholesterolemia, if despite maximal dietary and drug therapy LDL-cholesterol cannot be reduced sufficiently (documented for 12 months). No specific threshold is given because the overall risk profile of the patient should be considered in evaluating the indication for apheresis. It is however required that PCSK9 inhibitors are given before the patient is evaluated for apheresis [12]. Other countries have less specific recommendations with respect to apheresis for elevated LDL-cholesterol [5]. Generally, homozygous FH is widely recognized as an indication, while other forms of LDL-hypercholesterolemia are not.

## Apheresis for Elevated Lipoprotein(a)

Apheresis decreases the concentration of all apoB containing lipoproteins. Therefore, lipoprotein(a) concentrations are decreased to a similar extent as LDL. The role of apheresis in the treatment of elevated lipoprotein(a) is, however, much less well defined (compared to its role in treating LDL-hypercholesterolemia).

Only in Germany elevated Lipoprotein(a) levels are under certain conditions considered to be an indication for regular apheresis. According to German guidelines, apheresis may be indicated if lipoprotein(a) is > 60 mg/dl in patients with progressive cardiovascular disease despite optimal management of all other risk factors including LDL-cholesterol; either clinical progression or progression documented with imaging techniques is mandatory [13].

The National Lipid Association Expert Panel on familial hypercholesterolemia recommends apheresis in functional heterozygotes with LDL-cholesterol > 200 mg/dL (or non-HDL-cholesterol > 230 mg/dL) and additional risk factors which includes elevated lipoprotein(a) > 50 mg/dL though the FDA does not comment on apheresis for isolated elevated lipoprotein(a) [14]. Similarly, the HEART-UK criteria for the use of LDL apheresis include patients with progressive coronary artery disease, hypercholesterolemia, and lipoprotein(a) > 60 mg/dL in whom LDL-cholesterol remains elevated despite drug therapy [15].

In summary, Germany is the only country where apheresis may be considered for isolated lipoprotein(a) elevation. Other countries take elevated lipoprotein(a) as an additional risk factor into account when apheresis is considered for the treatment of LDL-hypercholesterolemia.

## Lipoprotein Apheresis Procedures

Previous reviews have summarized the different procedures used to perform lipoprotein apheresis [16]. Four of five available systems eliminate apoB containing particles, such as IDL, LDL, and lipoprotein(a). These lipoproteins are eliminated almost completely when passing through the filter or adsorption columns while VLDL, chylomicrons, and chylomicron remnants are eliminated to a lesser extent, because apoB is “protected” from binding in these larger lipoproteins. With these four systems, the elimination of LDL and lipoprotein(a) is very similar [16]. In contrast, the 5th system (Lipopac) eliminates specifically lipoprotein(a) and does not decrease levels of other apoB containing lipoproteins [17].

Observational data on lipoprotein apheresis favor regular treatment every week or every 2 weeks independent of the indication for treatment (elevated LDL and/or elevated lipoprotein(a)). The length of an individual apheresis session (between 1.5 and 4 h) depends on the plasma volume to be treated which itself depends on the LDL and lipoprotein(a) concentrations. The main vascular approach for all apheresis techniques is veno-venous. Only very few patients need shunt surgery. Some form of anticoagulation is mandatory for every apheresis system. A blood volume of approximately 500 ml circulates extracorporally. This can result in a drop in blood pressure. Anemia and iron deficiency are other associated side effects in some patients and may necessitate iron substitution if apheresis is conducted on a regular basis. However, generally speaking, all apheresis methods are tolerated well.

## The Effect of Apheresis on Atherosclerosis

The goal of apheresis is to decrease the risk for cardio-vascular events by reducing the plasma concentration of atherogenic lipoproteins, particularly LDL and lipoprotein(a). Although a number of studies have evaluated the effect of apheresis on lipid values, no adequately controlled and powered trial has been performed to test the hypothesis that apheresis reduces cardio-vascular event rates. However, there is a number of studies evaluating potential benefits of apheresis (Table 1).

**Table 1:** Studies evaluating lipoprotein apheresis on cardiovascular outcome (selected studies)

Study	Design	Population	Duration	Baseline LDL	Baseline Lp (a)	PE	Finding	Comment
Mabuchi [18]	Retrospective analysis; control group; not randomized	87 drug therapy; 43 drug therapy + apheresis	6 years	7.42 ± 1.73 (− 58% apheresis) 6.03 ± 1.32 (− 28% drugs)	Not indicated	Cardio-vascular events	72% lower event rate in apheresis vs drugs only (10% vs. 36%)	Strength: long-term; large study Limitation: not randomized; not blinded; different LDL levels in groups
Thompson [19]	Randomized, prospective, controlled (simvastatin + apheresis vs simvastatin + colestipol)	*N* = 39; 72% male he FH; CHD	2.1 years	6.5 ± 2.0 mmol/l	41 ± 15 μmol/l	Angio-graphic changes	No significant difference between drug only and apheresis group	Strength: prospective, randomized, controlled Limitation: Rel. low baseline lipoprotein(a) concentration; apheresis only every 2 weeks
Safarova [20]	Randomized, prospective, controlled (atorvastatin + apheresis vs. atorvastatin alone)	*N* = 30; 70% male; CHD; low LDL-chol (< 2.5 mmol/l); Elevated Lipoprotein(a) (> 50 mg/dl)	18 months	2.2 ± 0.2 mmol/l	102 ± 37 mg/dl	Angio-graphic changes	Significantly more regression and less progression with apheresis compered to atorvastatin alone	Strength: prospective, randomized, controlled; specific lipoprotein(a) elimination Limitations: small patient group; no clinical end points
Safarova [21]	Retrospective case series	*N* = 10; 70% female; 60 ± 9 years; severe hypercholesterolemia	10 ± 4 years	214 mg/dl (145;248)	26 mg/dl (15;109) 40% Lp(a) > 60 mg/dl	Change in Intima Media Thickness	Number of patients with CIMT > their "vascular age" decreased from 80 to 30%	Strength: long time course Limitations: small study; no control group; no clinical EP
Khan [22••]	Randomized, controlled cross over with sham apheresis	*N* = 20; refractory angina; lp(a) > 50 mg/dl	3 months	2.16 ± 0.73 mmol/l	110 mg/dl [77,159]	Myocardial perfusion reserve (MRI)	MPR, increased (0.47; 95% CI 0.31–0.63) compared with sham (-0.16; 95% CI—0.33–0.02) P < 0.001	Strength: controlled (sham apheresis); randomized Limitations: small study; short duration; no atherosclerotic end-points
Jaeger [23]	Retrospective analysis of events observed in periods before and after initiation of apheresis	*N* = 120; 72% male CHD; elevated lipoprotein(a) (> 2.14 μmol/l)	5.6 ± 5.8 years before initiation of apheresis vs 5.0 ± 3.6 years after initiation of apheresis	3.26 ± 1.27 mmol/l	4.21 ± 1.5 μmol/l	Change in event rate	Significant reduction of event rate: 1.056 per patient year vs. 0.144 per patient year	Strength: large number of patients; different apheresis systems Limitations: no control group; patients in the studies by Jaeger, Leebmann and Rosada overlap
Leebmann [24]	Retrospective analysis of events observed in periods before and after initiation of apheresis	*N* = 170; 72% male; progressive CHD despite low LDL-chol Elevated lipoprotein(a) (> 60 mg/dl)	2 years before initiation of apheresis vs 2 years after initiation of apheresis	2.56 ± 0.98 mmol/l	3.94 ± 1.77 μmol/l	Change in event rate	Significant reduction of event rate: 0.41 per patient year vs. 0.09 per patient year	Strength: large number of patients; only patients with elevated lipoprotein(a); different apheresis systems Limitations: no control group; patients in the studies by Jaeger, Leebmann and Rosada overlap
Rosada [25]	Retrospective analysis of events observed in periods before and after initiation of apheresis	*N* = 37; 95% male; progressive CHD despite low LDL-chol Elevated Lipoprotein(a) (> 60 mg/dl)	Event-free survival after first event vs. event-free survival after initiation of apheresis	2.18 ± 0.56 mmol/l	112 ± 34 mg/dl	1 year event-free survival rate	38% per year before initiation of apheresis vs. 75% per year after initiation of apheresis	Strength: only patients with elevated lipoprotein(a); different apheresis systems Limitations: no control group; patients in the studies by Jaeger, Leebmann and Rosada overlap
Moriarty [26]	Retrospective analysis of events observed in periods before and after initiation of apheresis	*N* = 14; progressive CHD despite low LDL-chol; Elevated lipoprotein(a) (> 60 mg/dl)	2 years before initiation of apheresis vs 2 years after initiation of apheresis	80 mg/dl	138 mg/dl	Change in event rate	94% reduction in major adverse cardiovascular events over a mean treatment period of 48 months	Limitations: no control group; small study

In a non-randomized trial, it was shown that patients on regular apheresis had lower LDL-cholesterol values and less cardio-vascular events than the control group treated by drug therapy alone [18]. In 2017, it was shown that significantly less vein graft occlusions (14.3% vs 27.4%) and progression of atherosclerosis occurred in patients treated with apheresis instead of statin treatment alone for the first year after coronary artery bypass. There were even signs of regression in the apheresis group. LDL-cholesterol and lipoprotein(a) were reduced by 50% [27].

In another trial, it was evaluated whether in patients with heterozygous familial hypercholesterolemia and coronary heart disease (CHD) (*n* = 39) biweekly apheresis in combination with simvastatin (40 mg/d) is superior to the simvastatin (40 mg/d) in combination with colestipol (20 g/d) [19]. After 2.1 years, there was no significant difference in angiographic changes between the two groups. The authors concluded that “decreasing lipoprotein(a) seems to be unnecessary if LDL-cholesterol is reduced to 3.4 mmol/l or less.” As the study did not select patients with elevated lipoprotein(a) concentrations, it is limited by a low baseline lipoprotein(a) concentration (43 mg/dl) and only a modest lipoprotein(a) reduction with apheresis (mean interval concentration 33 mg/dl) due to the biweekly apheresis interval. Finally, in an angiographic trial, it was evaluated whether atorvastatin together with specific lipoprotein(a) apheresis (lipopac apheresis) reduces CHD progression compared to atorvastatin alone in patients (*n* = 30) with CHD and elevated lipoprotein(a) (> 50 mg/dl) [20]. After 18 months patients treated with atorvastatin and apheresis compared to atorvastatin alone showed significantly more regression and less progression. Again, the trial is limited by a small number of subjects and the lack of reporting of clinical events. A long-term study evaluating the effect of 10 ± 4 years of apheresis on carotid intima media thickness (CIMT) recently showed that the number of patients with CIMT above their “vascular age” decreased from 80 to 30% over the treatment course — again without control group [21].

The most important (and convincing) study concerning apheresis for elevated lipoprotein(a) levels was published in 2017 [22••]. In a randomized, double blind, sham-apheresis controlled study design in 20 subjects with refractory angina and lipoprotein(a) > 50 mg/dl, it was shown that 3 months of weekly apheresis improved myocardial perfusion reserve and also exercise capacity.

Finally, there are a number of registry data indicating that the cardio-vascular event rate is lower in the time period following the initiation of regular apheresis compared to the time period before starting regular apheresis [23–26, 28]. All of these studies show a dramatic decrease in the event rate after initiation of regular apheresis (even more than 90% reduction in event rate). These evaluations are severely limited due to the lack of a control group. Progression of disease and thus recurrence of events are the main reasons for initiating apheresis. It is therefore not surprising to observe a very high event rate in the period preceding the initiation of apheresis. As outlined elsewhere, it is impossible to determine the true effect of apheresis without an adequate control group [29]. However, these registry data also show that side effects occurred in only 5% [30•].

## Conclusion

Regular lipoprotein apheresis remains a form of last resort therapy for very severe forms of hyperlipoproteinemia. It plays a role in the management of patients with severe LDL-hypercholesterolemia resistant to drug therapy (typically patients with homozygous familial hypercholesterolemia or severe heterozygous familial hypercholesterolemia) and in selected patients with elevated lipoprotein(a). Apheresis leads to a decrease in lipoprotein concentrations acutely by approximately 65%, which translates into significant interval mean reduction (30–50%). The treatment is tolerated well with minimal side effects though costs and time must be taken into account. Retrospective data indicates clinical benefit in patients undergoing regular apheresis therapy, but adequate, randomized controlled trials evaluating clinical outcome are lacking. Considering the development of new drugs, the future role of apheresis remains to be determined.
